# Pharmacokinetic and safety profile of tofacitinib in children with polyarticular course juvenile idiopathic arthritis: results of a phase 1, open-label, multicenter study

**DOI:** 10.1186/s12969-017-0212-y

**Published:** 2017-12-28

**Authors:** Nicolino Ruperto, Hermine I. Brunner, Zbigniew Zuber, Nikolay Tzaribachev, Daniel J. Kingsbury, Ivan Foeldvari, Gerd Horneff, Elzbieta Smolewska, Richard K. Vehe, Anasuya Hazra, Rong Wang, Charles A. Mebus, Christine Alvey, Manisha Lamba, Sriram Krishnaswami, Thomas C. Stock, Min Wang, Ricardo Suehiro, Alberto Martini, Daniel J. Lovell, for the Pediatric Rheumatology International Trials Organization (PRINTO), the Pediatric Rheumatology Collaborative Study Group (PRCSG)

**Affiliations:** 1 Istituto Giannina Gaslini, Clinica Pediatrica e Reumatologia, PRINTO, Genoa, Italy; 2 Cincinnati Children’s Hospital Medical Center, PRCSG, Cincinnati, OH USA; 3 St Louis Children’s Hospital ODS Rheumatology and Neurology, Krakow, Poland; 4 Pediatric Rheumatology Research Institute, Bad Bramstedt, Germany; 5 Randall Children’s Hospital, Portland, OR USA; 6 Hamburger Zentrum für Kinder- und Jugendrheumatologie, Hamburg, Germany; 7 Centre of General Pediatrics and Neonatology, Asklepios Klinik, Sankt Augustin, Germany; 8 Department of Pediatric Rheumatology, Medical University of Lodz, Lodz, Poland; 9 University of Minnesota Masonic Children’s Hospital, Minneapolis, MN USA; 10 Pfizer Inc, Collegeville, PA USA; 11 Pfizer Inc, Groton, CT USA; 12 Istituto Giannina Gaslini, Direzione Scientifica, Genoa, Italy

**Keywords:** Janus kinase inhibitor, Juvenile idiopathic arthritis, Tofacitinib, Pediatric, Pharmacokinetics, Safety, Dosing

## Abstract

**Background:**

Juvenile idiopathic arthritis (JIA) is the most common pediatric rheumatic disease and a leading cause of childhood disability. The objective of this study was to characterize the PK, safety, and taste acceptability of tofacitinib in patients with JIA.

**Methods:**

This Phase 1, open-label, multiple-dose (twice daily [BID] for 5 days) study of tofacitinib in patients with active (≥ 5 joints) polyarticular course JIA was conducted from March 2013–December 2015. Patients were allocated to one of three age-based cohorts: Cohort 1, 12 to < 18 years; Cohort 2, 6 to < 12 years; and Cohort 3, 2 to < 6 years. Tofacitinib was administered according to age and body weight as tablets or oral solution (grape flavor). PK were assessed on Day 5; safety was assessed at screening, Day 1, and Day 5. Taste acceptability of the oral solution was evaluated.

**Results:**

Twenty-six patients (age range 2–17 years) were enrolled: Cohort 1, *N* = 8; Cohort 2, *N* = 9; Cohort 3, *N* = 9; median tofacitinib doses were 5.0, 2.5, and 3.0 mg BID, respectively. The higher median tofacitinib dose in Cohort 3 versus Cohort 2 reflected implementation of an amended dosing scheme following an interim PK analysis after Cohort 2 recruitment. Geometric mean AUC at steady state (AUC_tau_) was 156.6 ng•h/mL in Cohort 1, 118.8 ng•h/mL in Cohort 2, and 142.5 ng•h/mL in Cohort 3; C_max_ (ng/mL) was 47.0, 41.7, and 66.2, respectively. C_trough_, C_min_, and t_1/2_ were similar in Cohorts 2 and 3, but higher in Cohort 1. Median time to C_max_ (T_max_) was similar between cohorts. Apparent clearance and volume of distribution decreased with decreasing age. Tofacitinib was well tolerated, with no serious adverse events or discontinuations due to adverse events reported. Taste acceptability was confirmed.

**Conclusions:**

PK findings from this study in children with polyarticular course JIA established dosing regimens and acceptable taste for use in subsequent studies within the tofacitinib pediatric development program.

**Trial registration:**

ClinicalTrials.gov: NCT01513902.

**Electronic supplementary material:**

The online version of this article (10.1186/s12969-017-0212-y) contains supplementary material, which is available to authorized users.

## Background

Juvenile idiopathic arthritis (JIA) is defined as arthritis of unknown etiology that presents in patients aged ≤16 years and persists for more than 6 weeks after other conditions have been excluded. According to the International League of Associations for Rheumatology classification [1, 2], JIA comprises seven mutually exclusive categories: systemic arthritis, persistent oligoarthritis or extended oligoarthritis, polyarthritis rheumatoid factor (RF)-negative, polyarthritis RF-positive, psoriatic arthritis, enthesitis-related arthritis, and undifferentiated arthritis.

JIA is the most common pediatric rheumatic disease, with an estimated incidence of approximately 8.2/100,000 in Europe [3] and 11.7–11.9/100,000 in the United States [4, 5]; it is one of the leading causes of disability over the short- and long-term [6]. Patients with a polyarticular course of JIA have involvement of ≥ 5 active joints during the first 6 months of disease [7].

Recommended pharmacologic treatment for patients with polyarticular course JIA may include initiation of nonsteroidal anti-inflammatory drugs (NSAIDs) for early disease [8]. Subsequent to this, and for patients with moderate or high disease activity, treatment with corticosteroids, non-biologic disease-modifying antirheumatic drugs (DMARDs; including methotrexate and leflunomide), and biologic DMARDs (including tumor necrosis factor inhibitors and those with a mechanism of action via interleukin-1, interleukin-6, and cytotoxic T-lymphocyte-associated protein-4) are recommended [8–12]. The course of treatment depends on disease severity, clinical features, and presence of prognostic factors [13].

Tofacitinib is an oral Janus kinase inhibitor approved for the treatment of rheumatoid arthritis (RA). Previous pharmacokinetics (PK) studies in adult patients with RA, and in healthy volunteers, have shown that tofacitinib is rapidly absorbed and eliminated. Systemic exposure to tofacitinib increases in an approximate dose-proportional manner following oral administration, with a time to peak plasma concentration (T_max_) of 0.5–1 h and a terminal phase half-life (t_1/2_) of approximately 3 h [14–17]. Following multiple dosing, steady state is achieved within 24–48 h with minimal accumulation.

A grape-flavored liquid formulation of tofacitinib has been developed for young children. In combination with safety data from the adult development program, the pediatric plan is intended to support the use of tofacitinib for the treatment of pediatric patients with polyarticular course JIA and systemic JIA.

The purpose of this Phase 1 study was to characterize the 5-day PK and short-term safety of tofacitinib following twice daily (BID) oral administration of multiple doses in children with active polyarticular course JIA. In addition, a further aim of this study was to evaluate the taste acceptability of the oral solution of tofacitinib.

## Methods

### Study design

This was a Phase 1, open-label, non-randomized, multicenter, multiple-dose study of tofacitinib (BID for 5 days) (A3921103; ClinicalTrials.gov: NCT01513902) in pediatric patients with JIA, conducted at nine Pediatric Rheumatology International Trials Organization and Pediatric Rheumatology Collaborative Study Group centers [18] in Germany, Poland, Slovakia, and the United States between March 2013 and December 2015.

### Patients

For inclusion in this study, eligible patients were aged 2 to <18 years and had active polyarticular course JIA (extended oligoarthritis, RF-positive or -negative, psoriatic arthritis or enthesitis-related arthritis) with ≥ 5 active joints at the time of enrollment, according to the American College of Rheumatology (ACR) definition of active joints.

Patients were excluded from the study if they had a diagnosis of systemic JIA, persistent oligoarthritis or undifferentiated JIA, a history of any other rheumatic autoimmune disease, or any condition affecting drug absorption.

Eligible patients were allocated to one of three cohorts based on their age: Cohort 1, aged 12 to <18 years; Cohort 2, aged 6 to <12 years; and Cohort 3, aged 2 to < 6 years. Recruitment of patients for this study was performed using a staggered sequential approach commencing with Cohort 1. Recruitment into Cohort 2 began after PK/safety data for the first four patients in Cohort 1 were reviewed, and recruitment for Cohort 3 began after reviewing PK/safety data for all enrolled patients in Cohorts 1 and 2. As a protocol amendment, an unplanned interim analysis was conducted (February 2015) following completion of all patients from Cohort 1 and 8 patients from Cohort 2.

All patients had the option to enter a Phase 3, open-label, long-term extension study evaluating the safety of tofacitinib (A3921145; ClinicalTrials.gov: NCT01500551). In addition to other criteria, to be eligible for the long-term extension study, patients had to have completed participation in this qualifying study of tofacitinib for the treatment of JIA and had to have sufficient evidence of JIA disease activity to warrant further use of tofacitinib (see Additional file 1).

### Treatment

Doses of tofacitinib were initially based on adult PK data and allometric (weight-based) scaling of these PK parameters. Tofacitinib doses were administered according to the age and body weight of the patient. In children aged between 2 to < 6 years weighing < 30 kg, and in those aged between 6 to < 18 years weighing < 40 kg, an oral solution was used. Tablets (7.9 mm round, deep biconvex shaped) were supplied to children aged 6 to < 18 years weighing ≥ 40 kg; however, an oral solution (1 mg/mL suspension) of tofacitinib was offered to children if this was their preference over tablets.

The initial dosing scheme is shown in the Additional file 1: Figure S1, and was based on the results of modeling.

On Days 1–4, tofacitinib was administered orally BID, with or without food, once in the morning and once in the evening, approximately 12 (±2) hours apart. On Day 5, only the morning dose was received, such that each patient received a total of nine doses of tofacitinib. The morning dose on Days 1 and 5 was administered at the study site; all other doses were administered at home by the patient or their guardian, following the dosing instructions provided to them.

Patients continued to receive their stable background treatment for JIA. Patients were permitted to receive concomitant treatment with methotrexate (maximum 20 mg/week or 15 mg/m^2^/week, whichever was lower) administered for at least 4 months with stable dosing for ≥ 6 weeks. Daily doses of NSAIDs and systemic corticosteroids (≤ 0.15 mg/kg/day, prednisone or equivalent) must have been stable for ≥ 4 weeks prior to administration of study drug. Treatment with the following pharmacologic therapies was not permitted during the study and had to be discontinued for specific time periods prior to the study: anakinra and etanercept ≥ 4 weeks; adalimumab ≥ 6 weeks; infliximab ≥8 weeks; golimumab ≥ 10 weeks; abatacept, tocilizumab, and certolizumab pegol ≥ 12 weeks; canakinumab ≥ 18 weeks; and ultraviolet phototherapy (≥ 2 weeks). Use of potent and moderate cytochrome P450 3A enzyme inhibitors and inducers was not permitted [15].

### Assessments

#### Pharmacokinetics

To characterize the PK of tofacitinib in plasma following multiple oral doses, blood samples (1.2 mL) for PK measurements were collected on Day 5 (morning) pre-dose and 0.5, 1, 2, 4, and 8 h after administration of tofacitinib. Blood was collected into lithium heparin tubes. To minimize discomfort of the patients during blood sampling, indwelling catheters may have been used and topical anesthesia may have been applied. Plasma was extracted (centrifugation at approximately 1700×*g* for 10 min at 4 °C) and stored at −20 °C within 1 h of collection.

Samples were analyzed at WuXi AppTec, Shanghai, China, using a validated analytical method in compliance with sponsor standard operating procedures. Tofacitinib samples were assayed using a high-performance liquid chromatography tandem mass spectrometric method. Calibration standard responses were linear over the range of 0.100–350 ng/mL, and the lower limit of quantification for tofacitinib was 0.100 ng/mL. The between-day assay accuracy, as a percent relative error, ranged from −1.5% to 1.7%, and the assay precision, expressed as the between-day percent coefficient of variation (%CV) was ≤ 5.9%.

PK parameters of tofacitinib were calculated using non-compartmental analysis of plasma concentration-time data using an internally developed and validated software system, eNCA (version 2.2.4). The PK parameters determined for tofacitinib were: AUC_tau_ (area under the plasma concentration–time curve from time zero to time tau [τ] – the dosing interval – where τ is equal to 12 h); C_max_ (maximum observed plasma concentration during the dosing interval); T_max_; t_½_; CL/F (apparent systemic clearance); V_z_/F (apparent volume of distribution); C_trough_ (trough [pre-dose] concentration); and C_min_ (lowest observed plasma concentration during the dosing interval).

The pre-dose (0 h) concentration was also used at 12 h, the end of the BID dosing interval, for PK parameter calculations, unless the observed 8-h concentration was lower than the pre-dose concentration. This was supported by the short PK t_½_ (approximately 3 h) of tofacitinib, which leads to achievement of steady state within 24 h of BID dosing. If the observed 8-h concentration was lower than the pre-dose concentration, then t_½_ was used to extrapolate from 8 to 12 h to determine AUC_tau_.

#### Safety

Adverse events (AEs) were assessed on Day 1 and Day 5 and were coded according to Medical Dictionary for Regulatory Activities (version 18.1) terms. Any serious AEs or deaths were to be reported at any time during the study.

Vital signs (blood pressure, pulse rate, and temperature), and laboratory data (hematology, chemistry, and urinalysis) were assessed at screening, Day 1, and Day 5. Patients underwent a complete physical examination at each visit.

#### Taste acceptability

A secondary objective of the study was to evaluate the taste acceptability of the grape-flavored solution formulation of tofacitinib. The taste assessment was only performed for patients who received the oral solution formulation of tofacitinib and utilized both proxy- and self-reported taste assessment questionnaires following dosing on Day 1. A parent-completed questionnaire was collected on Day 5 from a parent/guardian to obtain feedback on dosing at home during the outpatient period. Individuals completing the questionnaire were asked to rate the taste of the oral solution as ‘dislike very much’, ‘dislike a little’, ‘not sure’, ‘like a little’, or ‘like very much’.

#### Efficacy assessment

Efficacy parameters were collected and JIA response to tofacitinib was determined from Day 1 of the study. Specifically, six JIA core set measures were collected: the physician’s global assessment of JIA disease activity (rated 0–10; 0 = inactive); number of joints with active arthritis; number of joints with limitation of motion; C-reactive protein (CRP) levels; patient assessment of overall well-being (rated 0–10; 0 = very well); and physical function. The cross culturally adapted and validated version of the Childhood Health Assessment Questionnaire (CHAQ; rated 0–3; 0 = no disability), for each country participating in the study, was completed [19] to assess physical function.

Given the short duration of the PK phase, efficacy data are not reported here.

### Statistical analysis

The sample size for this study was determined using clinical trial simulations. The sample size of 24 pediatric patients (8 per age group) provided 80% probability that the ideal recommended dose resulting from the clearance estimate would have achieved a systemic exposure within 66% to 150% of the targeted exposure (5 mg BID), assuming an average adult CL/F of 20 L/h/70 kg, an estimated variability of 10% inter-study variability, an inter-individual variability of 40%, and a pediatric CL/F variability associated with non-compartmental analysis of 20%. The relationship between body weight and CL/F was simulated by the allometric power model, with an assumed power coefficient of 0.75, where an average body weight of 70 kg for an adult and body weight range of 7 to 85 kg for the pediatric patients was used in the simulation.

The PK analysis population was defined as all patients enrolled and treated who had at least one of the PK parameters of primary interest. For this population, plasma PK parameters from non-compartmental analysis (CL/F, V_z_F, AUC_tau_, C_max_, T_max_, and t_1/2_ [if data permitted]) on Day 5 were summarized descriptively by age group, gender, and race (i.e., each of these factors individually and overall). The actual dose was summarized descriptively by age group and overall. Plasma concentrations were summarized descriptively by age group and PK sampling time.

The linear regression between natural log-transformed apparent systemic CL/F against log-transformed body weight was performed in SAS® (version 9.2; SAS Institute Inc., Cary, NC, USA) to fit the power model on an untransformed scale: $CL(ped)/F=b0\times {\left(\frac{BWT}{70}\right)}^{b1}$, where b0 refers to the clearance in a patient weighing 70 kg and b1 is the exponent that determines the shape of the relationship between clearance and body weight; a value of 1 would indicate a linear relationship, and values < 1 a less than proportional change. The same log-linear regression between CL/F and body weight using baseline CRP levels, a biomarker for inflammation, as an additional covariate was also performed as an exploratory analysis.

All patients who received at least one dose of study drug were included in the safety analyses. Safety data were summarized descriptively.

Taste acceptability was summarized categorically (frequency and percentage) by age group for those patients who received the oral solution of tofacitinib.

## Results

### Patients

In total, 31 patients were screened; of these, 5 patients did not meet the entry criteria. Therefore, 26 eligible patients (age range 2–17 years) were enrolled in the study. All 26 patients completed the study and were evaluated for PK and safety. In addition, all patients subsequently enrolled in the open-label long-term extension study of tofacitinib.

Patient demographic and baseline disease characteristics are presented in Table 1. Baseline disease characteristics were generally similar across all cohorts, with the exception of body weight and height, and corresponding body mass index, which decreased from Cohort 1 (12 to < 18 years) to Cohort 3 (2 to < 6 years). More female than male patients were enrolled overall, and the proportion of female patients was higher in Cohort 3 compared with the other cohorts. All patients were white except for one patient in Cohort 2 (6 to < 12 years).

**Table 1:** Patient demographics and baseline disease characteristics

	Cohort 1 (12 to < 18 years) *N* = 8	Cohort 2 (6 to < 12 years) *N* = 9	Cohort 3 (2 to < 6 years) *N* = 9	All patients (2 to < 18 years) *N* = 26
Age at enrollment, years				
Median (Q1–Q3)	14.0 (12.0–16.0)	10.0 (8.0–11.0)	4.0 (4.0–5.0)	9.5 (5.0–12.0)
Gender, n (%)				
Female	5 (62.5)	5 (55.6)	7 (77.8)	17 (65.4)
Body mass index,^a^ kg/m^2^				
Median (Q1–Q3)	19.9 (17.1–21.9)	16.6 (15.9–20.4)	14.4 (14.3–16.2)	16.5 (15.0–20.2)
Height, cm				
Median (Q1–Q3)	168.8 (157.5–171.7)	135.0 (129.7–141.7)	109.7 (99.6–111.6)	134.5 (111.4–155.0)
Disease duration (years)				
Median (Q1–Q3)	1.2 (0.7–1.7)	0.5 (0.3–2.0)	2.8 (1.3–3.1)	1.4 (0.6–2.8)
*JIA categories*				
Polyarthritis (RF-)^b^, n (%)	6 (75.0)	7 (77.8)	9 (100.0)	22 (84.6)
Psoriatic arthritis, n (%)	1 (12.5)	1 (11.1)	0	2 (7.7)
Extended oligoarthritis, n (%)	1 (12.5)	1 (11.1)	0	2 (7.7)
*Disease characteristics*				
Physician’s global assessment of overall disease activity^c^				
Median (Q1–Q3)	7.5 (6.3–8.0)	8.0 (6.0–8.5)	6.5 (6.0–7.5)	7.3 (6.0–8.0)
Number of joints with active arthritis^c,d^				
Median (Q1–Q3)	15.5 (13.0–18.0)	17.0 (10.0–21.0)	10.0 (8.0–20.0)	14.0 (8.0–21.0)
Number of joints with limitation of motion^c^				
Median (Q1–Q3)	14.0 (8.5–16.5)	9.0 (8.0–15.0)	8.0 (6.0–20.0)	10.0 (7.0–17.0)
CHAQ functional ability				
Median (Q1–Q3)	NA	1.0 (0.4–1.6)	0.9 (0.6–1.5)	0.9 (0.4–1.6)
C-reactive protein, mg/L^e^				
Median (Q1–Q3)	0.3 (0.2–0.5)	0.6 (0.4–2.3)	2.2 (0.3–13.9)	0.5 (0.2–5.2)
*Concomitant drugs*				
Methotrexate use, mg/week				
n (%)	1 (12.5%)	0	8 (88.9%)	9 (34.6%)
Median dose (Q1–Q3)	25.0 (25.0–25.0)		10.0 (9.4–11.9)	10.0 (10.0–13.7)
Prednisone use, mg/day				
n (%)			2 (22.2%)	2 (7.7%)
Median dose (Q1–Q3)	0	0	3.5 (2.0–5.0)	3.5 (2.0–5.0)

*CHAQ* Childhood Health Assessment Questionnaire, *N* number of patients in each cohort, *NA* not applicable, *RF* rheumatoid factor, *SD* standard deviation

^a^BMI was calculated as weight in kg/(height in meters)^2^

^b^No patients with polyarthritis were RF+

^c^Baseline was defined as the Day 1 measurement for open-label long-term extension study

^d^Defined as a joint with swelling or a joint with pain/tenderness on movement

^e^Values < 0.2 mg/L were set to 0.1999 in summaries and analyses

Median CRP values were higher for patients in Cohort 3 compared with those in Cohorts 1 and 2. The median value for CRP remained within normal reference range (< 2.87 mg/L) across all cohorts.

The median number of joints with active arthritis across all patients was 14.0. Median disease duration from first onset was 1.4 years across all patients (quartile [Q] 1, Q3: 0.6, 2.8 years). Nine (9) patients (34.6%) across all cohorts received methotrexate as the concomitant drug during the study with a median dose of 10.0 mg/week. Of these, six patients received oral methotrexate.

### Summary of treatment administration

The median doses (mg BID) of tofacitinib (which were based on body weight) are presented in Table 2. Despite expected lower median body weight in Cohort 3 compared with the other groups, the increase in median BID dose of tofacitinib in Cohort 3 compared with Cohort 2 was due to the amended dosing scheme, following an interim analysis of PK data from Cohorts 1 and 2.

**Table 2:** Descriptive summary of plasma tofacitinib pharmacokinetic parameter values by cohort

Parameter	Cohort 1 (12 to < 18 years) *N* = 8^a^	Cohort 2 (6 to < 12 years) *N* = 9^b^	Cohort 3 (2 to < 6 years) *N* = 9	All patients (2 to < 18 years) *N* = 26^c^
Dose, median (range), mg BID	5.0 (3.0–5.0)	2.5 (2.0–5.0)	3.0 (2.5–3.5)	3.0 (2.0–5.0)
AUC_tau_, geometric mean (%CV),^d^ ng•h/mL	156.6 (25)	118.8 (27)	142.5 (32)	138.6 (30)
C_max_, geometric mean (%CV),^d^ ng/mL	47.0 (40)	41.7 (29)	66.2 (28)	50.7 (38)
T_max_, median (range), h	0.8 (0.5–6.9)	1.0 (0.5–2.1)	0.5 (0.5–1.9)	0.9 (0.5–6.9)
C_trough_, geometric mean, (%CV),^d^ ng/mL	2.7 (100)	0.8 (127)	0.8 (119)	1.1 (145)
C_min_, geometric mean, (%CV),^d^ ng/mL	2.5 (86)	0.8 (95)	0.7 (103)	1.1 (123)
t_½_, arithmetic mean (SD), h	2.6 ± 0.5	1.9 ± 0.3	1.8 ± 0.4	2.1 ± 0.5
CL/F, geometric mean, (%CV),^d^ L/h	28.1 (22)	25.5 (40)	20.5 (33)	24.3 (34)
V_z_/F, geometric mean, (%CV),^d^ L	104.9 (35)	71.0 (40)	51.4 (34)	70.5 (47)

*%CV* percent coefficient of variation, *AUC*_*tau*_ area under the plasma concentration–time curve from time zero to time tau, *CL/F* apparent systemic clearance, *C*_*max*_ maximum observed plasma concentration during the dosing interval, *C*_*min*_ lowest observed plasma concentration during the dosing interval, *C*_*trough*_ trough (pre-dose) concentration, *N* number of patients in each cohort, *SD* standard deviation, *t*_*1/2*_ terminal phase half-life, *T*_*max*_ time to peak plasma concentration, *V*_*z*_ */F* apparent volume of distribution

^a^ *N* = 7 for t_½_ and V_z_/F due to lack of a well-characterized terminal phase in 1 patient

^b^ *N* = 8 for t_½_, V_z_/F, CL/F, C_min_, and AUC_tau_ due to incomplete pharmacokinetics sampling for 1 patient

^c^ *N* = 24 for t_½_ and V_z_/F, and *N* = 25 for C_min_. AUC_tau_, and CL/F due to the exceptions noted above

^d^Geometric %CV

Overall, 6 patients in Cohort 1, and 2 patients in Cohort 2, received tofacitinib 5 mg BID as a tablet. All other patients received an oral solution of tofacitinib (Cohort 1, 3 mg BID [*n* = 2]; Cohort 2, 3 mg BID [*n* = 2], 2.5 mg BID [*n* = 3], and 2 mg BID [*n* = 2]; Cohort 3, 3.5 mg BID [*n* = 1], 3 mg BID [*n* = 6], and 2.5 mg BID [*n* = 2]).

### Pharmacokinetics

**Fig. 1:**
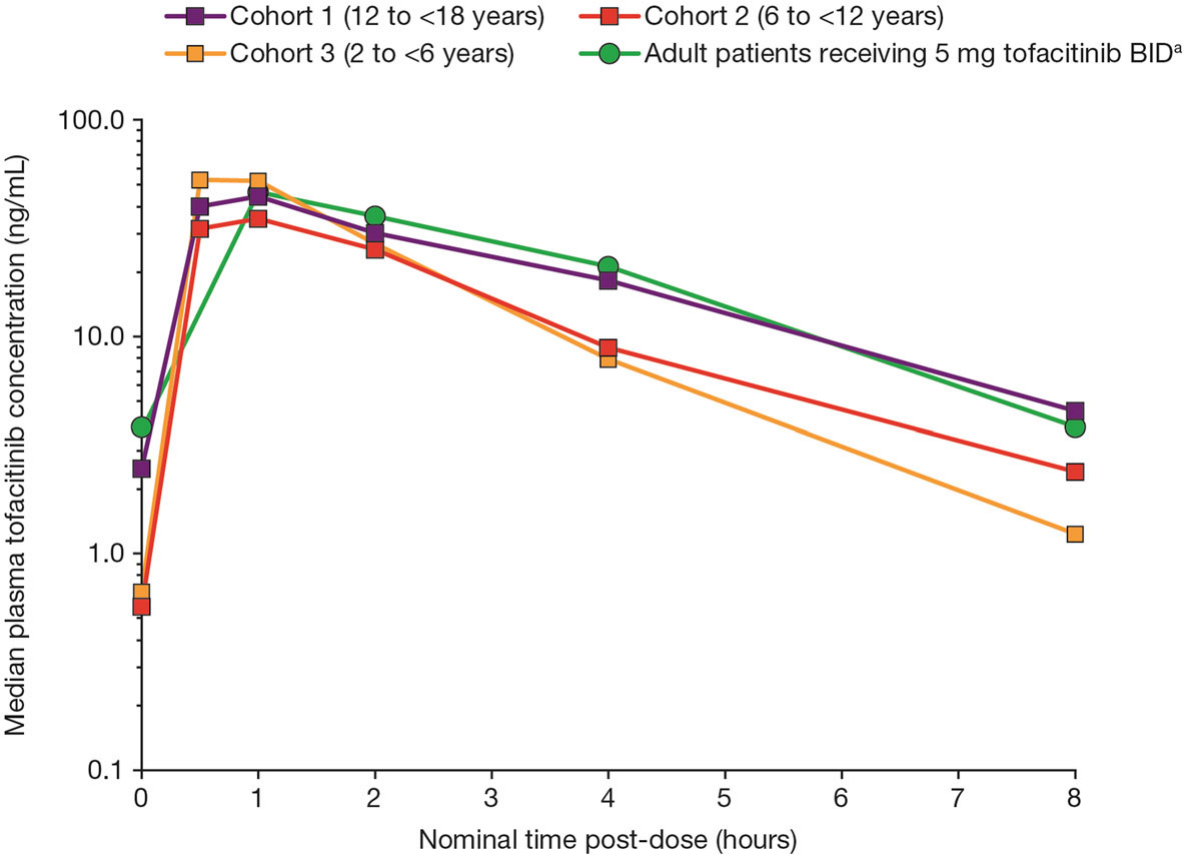
Median tofacitinib plasma concentration–time profiles for each cohort. ^a^Mean tofacitinib plasma concentration–time profile for adult patients who received tofacitinib 5 mg BID in a Phase 2 dose-ranging study in patients with active rheumatoid arthritis [20]. *BID* twice daily

Median tofacitinib plasma concentration–time profiles on Day 5 for each cohort (semi-logarithmic scale) are presented in Fig. 1 and PK parameters are summarized descriptively by cohort in Table 2. The mean tofacitinib plasma concentration–time profile for adult patients with RA who received tofacitinib 5 mg BID in a Phase 2 dose-ranging study is presented for comparison [20].

Geometric mean AUC at steady state (AUC_tau_) was lower in Cohort 2 (6 to < 12 years; 118.8 ng•h/mL) relative to that in Cohort 1 (12 to <18 years; 156.6 ng•h/mL) (Table 2) while the AUC_tau_ in Cohort 3 (2 to < 6 years; 142.5 ng•h/mL) was comparable with that in Cohort 1. C_max_ values of Cohorts 1 and 2 were similar, but C_max_ of Cohort 3 was higher than that of the other cohorts.

C_trough_ (pre-dose), C_min_, and t_1/2_ values were similar between Cohort 2 and Cohort 3, and were lower in these cohorts than those in Cohort 1. Median time to C_max_ (T_max_) values were similar across all three cohorts.

Geometric mean values for both apparent systemic clearance (CL/F) and apparent volume of distribution (Vz/F) decreased with decreasing age (Cohort 1 through 3). Variability (expressed as geometric %CV) for AUC_tau_, C_max_, CL/F, and Vz/F was ≤ 40%. The geometric %CV for C_trough_ and C_min_ ranged from 86% to ~145%, indicating high variability in these two parameters.

No clinically important gender differences were observed in any of the PK parameters (Additional file 1: Table S1). Given the racial make-up of the current study population (25/26 [96.2%] patients were white), the potential effect of race on the PK of tofacitinib was not evaluable.

**Fig. 2:**
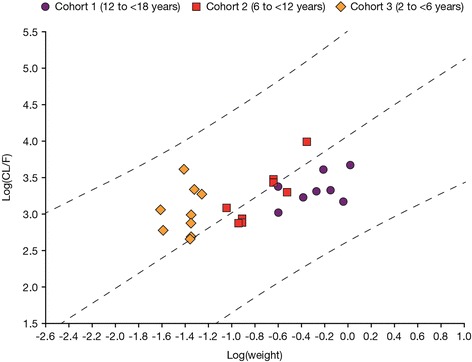
Log-linear regression analysis of individual CL/F (L/h) of plasma toficitinib versus body weight and 90% confidence region. Regression equation ln(CL/F) = 3.5018 + 0.370973 × ln(weight/70). Weight has been standardized to an average adult weight of 70 kg first, then log-transformed. *N = 8* for CL/F in Cohort 2 due to incomplete pharmacokinetics sampling for 1 patient. *CL/F* apparent plasma clearance

The log-linear regression analysis of individual CL/F (L/h) versus body weight of the patients resulted in a positive slope of 0.37 (90% confidence interval [CI]: 0.18–0.56), indicating that CL/F increased with an increase in body weight (Fig. 2). However, the estimated mean slope was considerably lower than the commonly used allometric exponent of 0.75 (to scale clearance from adults to children), which was used to derive the initial dosing scheme; the 90% CI of the slope excludes 0.75. Based on the estimated intercept (3.50) and slope (body weight) parameters (0.37), mean CL/F values for patients with body weights of 15, 25, 50, and 70 kg were estimated to be 18.7, 22.6, 29.3, and 33.2 L/h, respectively.

Consideration of baseline CRP of the patients, as a measure of inflammation, in addition to body weight in an exploratory log-linear regression analysis did not appear to have a notable effect on the relationship between body weight and CL/F of tofacitinib, resulting in a positive estimated mean slope of 0.33 (90% CI: 0.15–0.51) for body weight. From this exploratory analysis, CRP alone did not appear to have a significant effect on CL/F (estimated mean slope − 0.06 with 90% CI including 0).

### Safety

All 26 patients were evaluable for AEs; of these, 4 patients (2 patients in Cohort 3 [2 to < 6 years] and 1 patient each in Cohort 1 [12 to <18 years] and Cohort 2 [6 to <12 years]) reported a total of 4 AEs. In Cohort 1, fatigue was reported by 1 patient, and this AE was considered by the investigator to be related to study treatment. One patient in Cohort 2 reported an AE of anemia on Day 5 (hemoglobin values: 111, 102, and 98 g/L at screening, on Day 1, and on Day 5, respectively), and two patients in Cohort 3 reported AEs of viral infection and blister (on the toe) (1 patient each); none of these AEs were considered treatment related.

The AEs of anemia (Cohort 2), fatigue (Cohort 1), and blister (Cohort 3) were mild in severity, whereas the AE of viral infection (Cohort 3) was moderate in severity. AEs of fatigue and anemia were resolved during the study, whereas the AEs of blister and viral infection were unresolved at the end of the study, but subsequently resolved.

No serious AEs, severe AEs, or deaths were reported in this study, and no patients permanently or temporarily discontinued treatment, or reduced their tofacitinib dose, due to an AE during the study.

No clinically significant changes in laboratory test results from baseline were recorded. Other than those relating to the AE of viral infection, no vital signs results or physical examination findings were of clinical concern during the study.

### Taste evaluation

In total, 18 patients received the oral solution of tofacitinib: 2/8 (25.0%) patients in Cohort 1 (12 to <18 years); 7/9 (77.8%) patients in Cohort 2 (6 to < 12 years); and 9/9 (100.0%) patients in Cohort 3 (2 to < 6 years). Table 3 presents the categorical summary of the taste assessment on Day 1 and Day 5. Overall, patients found the grape-flavor taste of the study drug acceptable. In general, there were no differences in the assessment of taste of the tofacitinib oral solution between Day 1 and Day 5, with 11/18 (61.1%) and 9/18 (50.0%) patients, respectively, specifying that they liked the taste ‘a little’ or ‘very much’.

**Table 3:** Categorical summary for taste assessment (safety population)

		Taste assessment categories, n (%)				
	Day	Dislike very much	Dislike a little	Not sure	Like a little	Like very much
Cohort 1 (12 to < 18 years) *N* = 2	1	0	0	1 (50.0)	1 (50.0)	0
	5	0	1 (50.0)	1 (50.0)	0	0
Cohort 2 (6 to < 12 years) *N* = 7	1	1 (14.3)	0	1 (14.3)	3 (42.9)	2 (28.6)
	5	1 (14.3)	0	2 (28.6)	2 (28.6)	2 (28.6)
Cohort 3 (2 to < 6 years) *N* = 9	1	1 (11.1)	2 (22.2)	1 (11.1)	1 (11.1)	4 (44.4)
	5	0	3 (33.3)	1 (11.1)	2 (22.2)	3 (33.3)

*N* number of patients who completed the taste assessment, *n* number of patients with specified criteria

## Discussion

The findings of this Phase 1 trial suggest that the PK of tofacitinib (BID regimen) was adequately characterized in patients with polyarticular course JIA and these data support the use of a body weight-based dosing regimen in children with polyarticular course JIA. In the studied patients, tofacitinib was well tolerated, and the taste of the oral solution of tofacitinib was considered acceptable.

Doses of tofacitinib BID were selected based on allometric scaling of CL/F from adult patients with RA in order to achieve comparable efficacious exposures. However, interim analyses of PK data from the first 14 patients in this study indicated higher CL/F values when compared with historical data in adult patients with RA (18.4 L/h) [21]. This resulted in lower steady state AUC_tau_ and C_max_ values in children with JIA aged 6 years or more compared with adult patients with RA. Consequently, a new weight-based dosing scheme was implemented for younger children (< 6 years old) with increased tofacitinib doses from those initially planned, to enable achievement of exposures that are known to be efficacious in adult patients with RA (from a dose of approximately 5 mg BID tofacitinib).

Based on all available data from the study, the geometric mean tofacitinib apparent systemic clearance (CL/F) values in this study were higher across all age groups, compared with historical data in adult patients with RA receiving 5 mg BID dosing (18.4 L/h) [21]. The results from Cohort 3 indicate that the dose increase implemented, based on preliminary data from the earlier cohorts, resulted in systemic exposures that were comparable with those in adult patients with RA. Geometric mean values for both CL/F and apparent volume of distribution (Vz/F) decreased with decreasing age (Cohort 1 through 3), which could primarily be attributed to decreasing mean BMI with age. Median T_max_ values of tofacitinib in each cohort were comparable with that observed in adult patients with RA. Finally, mean t_1/2_ values for tofacitinib in the three cohorts were shorter than that observed in adult patients with RA (approximately 3 h) [14–17].

The efficacy and safety of tofacitinib 1–5 mg BID administered in tablet form (5 mg) or as an oral solution (1 mg/mL) are being evaluated in a 44-week, Phase 3, randomized withdrawal, double-blind placebo-controlled study in pediatric patients with JIA (A3921104; ClinicalTrials.gov: NCT02592434), which is utilizing suggested doses by weight as derived from this study. In addition, a Phase 3, open-label, follow-up, long-term extension safety study (A3921145; ClinicalTrials.gov: NCT01500551) is ongoing in pediatric patients with JIA who have previously participated in qualifying/index JIA studies of tofacitinib (including patients enrolled in the study presented here). A third study (Study A3921165; ClinicalTrials.gov: NCT03000439), of the efficacy, safety, and PK of tofacitinib 5 mg and 10 mg BID in tablet form or as an oral solution is also planned. This randomized withdrawal, double-blind placebo-controlled study will evaluate the time to flare in pediatric patients with systemic JIA, a type of JIA associated with symptoms of fever and rash.

In this study, tofacitinib administered BID as either 5 mg tablets, or a 1 mg/mL oral solution, over 5 days (range of median doses, 2.5–5.0 mg BID) was generally well tolerated in pediatric patients (from 2 to < 18 years). During the study, only one treatment-related AE was reported, and resolved without tofacitinib dose adjustment or discontinuation. In the clinical development program in adult patients with RA, the most commonly reported AEs were nasopharyngitis, upper respiratory tract infection, and urinary tract infection, and the most common System Organ Class of serious AEs was infections and infestations [22]. None of these AEs were reported in the current study. The taste of tofacitinib oral solution was generally acceptable to patients. All patients completed this study and enrolled in a long-term extension study, which is evaluating the safety of tofacitinib in pediatric patients.

Limitations of this study include the small number of patients enrolled and the short duration of exposure to tofacitinib, which allowed only short-term tolerability to be assessed and precludes any definitive conclusions on safety from being made. Also, the short duration of the study was not sufficient to allow efficacy findings to be inferred.

## Conclusions

In conclusion, the PK findings from this open-label Phase 1 study in pediatric patients with polyarticular JIA have allowed the establishment of tofacitinib dosing regimens for this patient population and enabled the assessment of efficacy and safety in Phase 3 studies within the tofacitinib pediatric development program. Tofacitinib was well tolerated and the taste was acceptable.

## Additional file

Additional file 1: Inclusion criteria for the long-term extension study, dosing scheme, and descriptive summary of plasma tofacitinib pharmacokinetic parameter values by gender. (PDF 39 kb)

## Abbreviations

%CV: Percent coefficient of variation
AE: Adverse event
AUC_tau_: Area under the plasma concentration–time curve from time zero to time tau
BID: Twice daily
CHAQ: Childhood Health Assessment Questionnaire
CI: Confidence interval
CL/F: Apparent systemic clearance
C_max_: Maximum observed plasma concentration during the dosing interval
C_min_: Lowest observed plasma concentration during the dosing interval
CRP: C-reactive protein
C_trough_: Trough (pre-dose) concentration
DMARD: Disease- modifying antirheumatic drug
JIA: Juvenile idiopathic arthritis
NSAID: Nonsteroidal anti-inflammatory drug
PK: Pharmacokinetics
RA: Rheumatoid arthritis
RF: Rheumatoid factor
t_1/2_: Terminal phase half-life
T_max_: Time to peak plasma concentration
V_z_/F: Apparent volume of distribution
τ: Tau
