# Supplementary material for: Pharmacokinetic and safety profile of tofacitinib in children with polyarticular course juvenile idiopathic arthritis: results of a phase 1, open-label, multicenter study
Source: Pediatr Rheumatol Online J. 2017 Dec 28;15:86. doi: 10.1186/s12969-017-0212-y (PMC5745974; doi:10.1186/s12969-017-0212-y)
Supplement: Additional file 1: — Inclusion criteria for the long-term extension study, dosing scheme, and descriptive summary of plasma tofacitinib pharmacokinetic parameter values by gender. (PDF 39 kb) [file 12969_2017_212_MOESM1_ESM.pdf]

Additional material

Inclusion criteria for the long-term extension study

1. Pediatric patients with juvenile idiopathic arthritis (JIA) aged from 2 to <18 years who met entry criteria for the qualifying/index study and, in the opinion of the investigator, have sufficient evidence of JIA disease activity to warrant use of tofacitinib as a disease-modifying antirheumatic drug. Patients turning 18 years of age during participation in the qualifying/index study or subsequently will be eligible for participation in this study.

2. The patient has discontinued disallowed concomitant medications for the required time prior to the first dose of study drug, and is taking only those concomitant medications in doses and frequencies allowed by the protocol.

3. Patients must have previously completed participation in a qualifying study of tofacitinib for the treatment of JIA. Patients who have required earlier discontinuation of treatment in a qualifying study for reasons other than tofacitinib-related serious adverse events may be eligible.

4. For patients receiving methotrexate treatment, methotrexate may be administered either orally or parenterally at doses not to exceed 20 mg/week or 15 mg/m^2/week, whichever is lower. Patients taking methotrexate must be taking folic acid or folinic acid in accordance with local standards.

5. For patients receiving an oral glucocorticoid, glucocorticoids may be administered at a maximum dose of 0.20 mg/kg/day or 10 mg/day, prednisone or equivalent, whichever is lower.

6. For patients receiving leflunomide treatment, leflunomide may be administered according to the following dosing scheme: 10 mg every other day for patients weighing less than 20 kg; 10 mg every day for patients weighing between 20 and 40 kg; 20 mg every day for patients weighing over 40 kg; or as according to local standards.

7. For patients receiving sulfasalazine, chloroquine, or hydroxychloroquine treatment, these medications may be administered according to local standards.

Additional Fig. 1 Dosing scheme

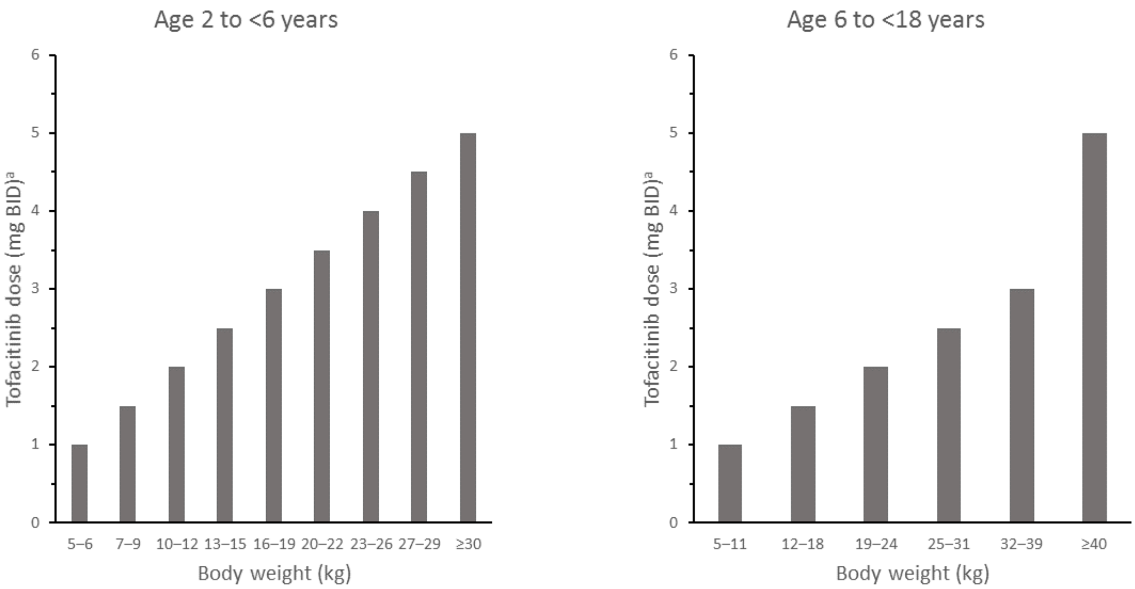

[a]All suspensions were administered at a concentration of 1 mg/mL

BID twice daily

Additional Table 1 Descriptive summary of plasma tofacitinib pharmacokinetic parameter values by gender

	**Male (*N* = 9)[a]**	**Female (*N* = 17)[b]**
**AUC_{tau}, geometric mean (%CV),[c] ng•h/mL**	140.4 (33)	137.7 (29)
**C_{max}, geometric mean (%CV),[c] ng/mL**	47.2 (21)	52.7 (45)
**T_{max}, median (range), h**	1.0 (0.5–2.1)	0.7 (0.5–6.9)
**C_{trough}, geometric mean, (%CV),[c] ng/mL**	1.1 (188)	1.1 (133)
**C_{min}, geometric mean, (%CV),[c] ng/mL**	1.3 (150)	1.0 (116)
**$t_{½}$, arithmetic mean ±SD, h**	2.3 ± 0.7	2.0 ± 0.4
**CL/F, geometric mean, (%CV),[c] L/h**	23.5 (41)	24.7 (32)
**V_z/F, geometric mean,[c] L**	74.1 (48)	68.8 (48)

[a]*N* = 8 for $t_{½}$, V_z/F, CL/F, C_{min}, and AUC_{tau} due to incomplete pharmacokinetics sampling for 1 patient

[b]*N* = 16 for $t_{½}$ and V_z/F due to lack of a well-characterized terminal phase in 1 patient

[c]Geometric %CV

%CV percent coefficient of variation; *AUC_{tau}* area under the plasma concentration–time curve from time zero to time tau; *CL/F* apparent systemic clearance; *C_{max}* maximum observed plasma concentration during the dosing interval; *C_{min}* lowest observed plasma concentration during the dosing interval; *C_{trough}* trough (pre-dose) concentration; *N* number of patients in each cohort; *SD* standard deviation;

$t_{1/2}$ terminal phase half-life; T_{max} time to peak plasma concentration; V_z/F apparent volume of distribution
